# Bio-Preparation and Regulation of Pyrrole Structure Nano-Pigment Based on Biomimetic Membrane

**DOI:** 10.3390/nano9010114

**Published:** 2019-01-18

**Authors:** Jixian Gong, Jiayin Liu, Xueqiang Tan, Zheng Li, Qiujin Li, Jianfei Zhang

**Affiliations:** 1 School of Textiles Science and Engineering, Tianjin Polytechnic University, Tianjin 300387, China; liujiayin6@126.com (J.L.); tan_xueqiang@126.com (X.T.); lizheng_nx@163.com (Z.L.); maldini@mail.nankai.edu.cn (Q.L.); 2 Key Laboratory of Advanced Textile Composites Ministry of Education, Tianjin Polytechnic University, Tianjin 300387, China

**Keywords:** transmembrane transport, biomimetic membrane, biomass pigments, nano-pigment, microbial pigments, lipid bilayers

## Abstract

Microbial pigments, regarded as the most potential biomass pigments, have lately attracted increasing attention in textile dyeing due to their sustainability and cleaner production. The pyrrole structure microbial pigment, called prodigiosin, recently have become a research hotspot for its bright colors and antibacterial function. However, in most case the extraction and preparation are time-consuming and expensive processes since these kinds of microbial pigments are intracellular metabolites. In order to promote the application of microbial pigments in textile dyeing, a novel idea of preparing dye liquid of pyrrole structure pigments based on fermentation broth was put forward via increasing the proportion of extracellular pigments. A model membrane platform was established with a planar lipid bilayer to investigate transmembrane transport of microbial pigments and permeability barrier of cell membrane. The nano-dispersion of pigments was produced as the dye liquor owing to high-throughput transmembrane transfer of intracellular pigments and the increase of extracellular pigments proportion. The results indicated that the size and surface electrical properties of the pigments had contributed much to the mass transfer. It is also showed that transmembrane transmission of the intracellular pigments could be regulated by physical and chemical methods. With the improvement of transmembrane transfer efficiency of microbial pigments and the proportion of extracellular pigments, the complicated biological separation process could be avoided and the application of microbial pigments in textile dyeing can be promoted.

## 1. Introduction

Cleaner production has become the theme of textile dyeing in recent years. Biomass pigments have attracted more attention in the dyeing process owing to their excellent characteristics such as sustainability, natural functionality and biocompatibility. Compared with insect and plant pigments, microbial pigments are more suitable for industrial production. Microbial pigments have excellent prospects for industrial application since the sources are not limited by seasonal, climatic, and geographical factors [1,2,3,4,5,6,7]. Microbial pigments are considered as a promising source for dyestuff of textile. The red pigment derived from organism is an important member in the family of dyestuff from biomass. Pyrrole structure microbial pigment, called prodigiosin, recently have become a research hotspot for its bright colors and antibacterial function [8,9]. This pigment was a kind of intracellular metabolite. And the pyrrole structure red pigment was mostly insoluble in water at room temperature. In general, pyrrole structure red pigments had to be extracted from the interior of thalli by organic solvent before preparing dye liquor. However, the use of organic solvents not only increased the cost, but also did not conform to the cleaner production [10,11].

An interesting phenomenon that has been found in our research of pyrrole structure red pigments produced by microorganisms is that the pigment synthesized in cells can be detected in the form of nanoscale particles in extracellular culture. This inspired a novel strategy for application of bio-pigment produced in cell as intracellular metabolite. If the transmembrane transport of pigments can be promoted by regulating cellular metabolism, the concentration of the extracellular pigments in the fermentation culture would be increased. The suspension of the pigment was quite stable as the pigment excreted out of the cell was insoluble and in nano-particle form. Thus, the dyeing liquid of microbial pigments could be prepared based on the fermentation broth. The extraction and separation of microbial pigments could be avoided, which was time-consuming and cost-consuming. Meanwhile, the dyeing cost of microbial pigments could be reduced. 

In this investigation, pyrrole structure pigment was produced by cell metabolization and presented in the form of nanoscale particles in the suspension for dyeing of textiles. Presently, studies about nano-pigment mostly focus on the synthesis of inorganic nanoparticles by chemical methods [12,13,14,15,16]. The report about the preparation of organic nanoparticles by biosynthesis is still rare [17]. 

To improve the proportion of pigment in culture, the trans-membrane behavior should be investigated. As the outermost boundary, the cell membrane plays an important role in vital activity of organisms. It can maintain a stable environment through the selective exchange inside and outside the cell. Since the mechanism of substance transport through cell membranes are complicated, a range of simplified model systems such as lipid vesicles in solution and black lipid membranes have been established to mimic the fundamental architecture of the membrane. Membrane mimetic chemistry is an interdisciplinary research based on chemistry, physics, and biology [18]. The research has attracted worldwide attention. Membrane mimetic chemistry can realize the preparation and kinetic study of membrane by the simulation of artificial membrane [19]. Many life phenomena can be studied using artificial membranes, such as substance transport [20,21], energy metabolism [22], cell recognition, nerve conduction, etc. 

Phospholipid bilayers are self-assembly, dynamic, and closely similar to cell membranes. It can be used for studies of trans-membrane transport in vitro, which is very difficult to achieve with real cell membranes. In this study, a mimic phospholipid bilayer membrane was constructed as an in vitro platform to research the trans-membrane behavior. For convenience, a sort of typical and classical particles, such as zinc ion, methylene blue, the chemical synthetic dye of acid orange was employed in this study to investigate the process of transmission of nanoparticles across membrane and to find the way to improve the transport efficiency [23,24]. Zinc ion is a typical material on researches of transmembrane mass transfer. Methylene blue, because of its simple molecular structure, is often used as a representative of dye in various studies. Acidic orange II molecule and methylene blue molecule are similar in size. Both of them had bright colors, which was beneficial for observation of experimental phenomena. 

It is generally believed that mass transfer performance of substances is mainly affected by their sizes, surface electrical properties, and hydrophilic and hydrophobic properties. In this investigation, the effect of particle characterization, such as the size and surface electrical properties, was studied on the transportation efficiency across the membrane. Moreover, the improvement of trans-membrane deliver with physical and chemical methods were also performed. 

**Figure 1:**
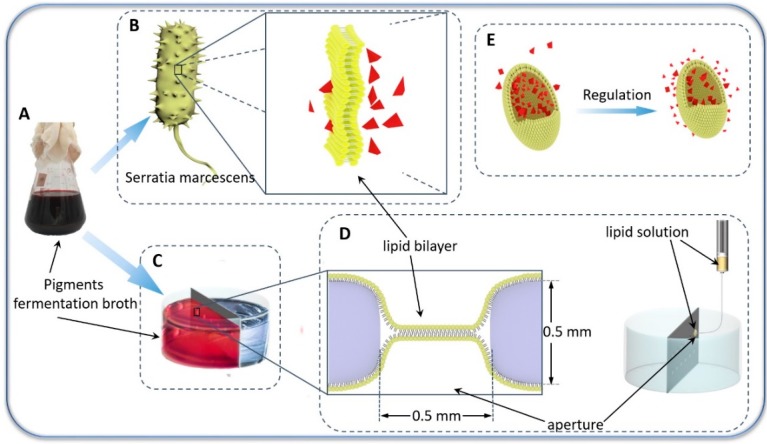
Schematic depiction of the biomimetic membrane platform for transmembrane transport studies of microbial pigments. (**A**) Pyrrole structure nano-pigments fermentation broth. (**B**) Schematic of *Serratia marcescens* (left) and lipid bilayers (right). (**C**) Schematic of pigments transmembrane transport experiment. (**D**) Preparation process of the biomimetic membrane. (**E**) Schematic illustration of intracellular pigment distribution.

On this basis, the proportion of extracellular pigments was regulated by controlling the permeability of the cellular membrane. For the first time, the biomimetic membranes were employed to study transmembrane transport of microbial pigments for preparation of dye liquor. As shown in Figure 1, the model membrane platform of lipid bilayer membrane was established. This research explored the breakthrough methods of cell membrane barrier and the biological preparation of microbial pigments based on the results of the biomimetic membrane experiment. Thus, the application of microbial pigments can be developed by efficient preparation of microbial dye liquor based on fermentation broth.

## 2. Materials and Methods

### 2.1. Materials

*Serratia marcescens* ATCC 8100 was purchased from American type culture collection.

Chemical and reagents: Standard prodigiosin was bought from Abcam Company, Cambridge, UK. Soy lecithin, cholesterol, peptone, beef extract, agar and yeast powder were of biological reagent. N-octane, Tween 80, Trition X-100, methylene blue, acid orange II, ZnCl_2_, KCl, NaCl, MgSO_4_, NaOH, ethanol, glycerol, and hydrochloric acid were of analytical reagent grade.

### 2.2. Preparation of Stable Phospholipid Bilayers

It was reported that the cell membrane was relatively stable when the ratio of lecithin to cholesterol was about 3:1 [25]. The experiments were carried out to regulate transmembrane transport of different size substances. In order to prepare a phospholipid bilayer having good stability and conductivity, 0.48 g lecithin and 0.16 g cholesterol were dissolved in 10 mL of n-octane and kept in the ultrasonic bath for 40 min. The film-forming solution could be used to prepare artificial membranes after being left to stand for 24 h.

**Figure 2:**
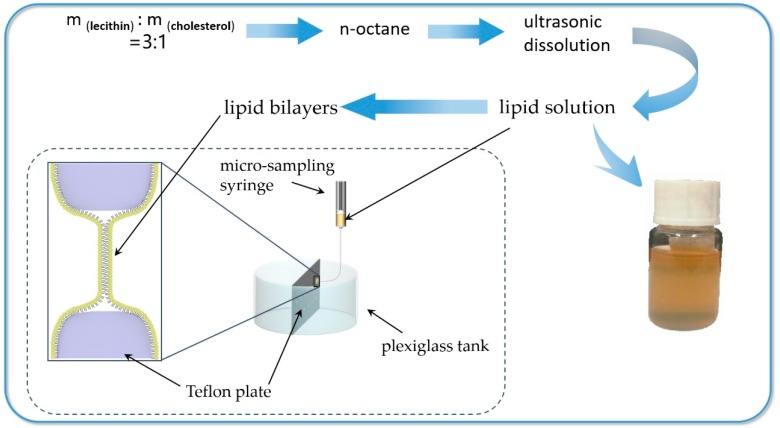
Flowchart for the Preparation of lipid bilayers.

The experiment was carried out using a Plexiglas tank which was divided into two rooms by a Teflon plate. The volume of the single room is 60 mL. There are 26 holes arranged on the plate, which are divided into upper and lower rows with a spacing of 5 mm and a diameter of 5 mm. The intracellular and extracellular environment were simulated by left and right rooms of adding the dye liquor, organic solvent or buffer solution. A small amount of the film-forming solution was taken up by a micro-sampling needle to drop into the small hole, then it was automatically dispersed to form a bilayer lipid membrane. At the same time, it was ensured that the dropped liquid level after sampling of 6 mL did not reach the small hole. The preparation of lipid bilayers can be summarized in form of a flowchart as shown in Figure 2.

### 2.3. Transmembrane Mass Transfer Experiment of Substances with Different Physicochemical Properties

As it has been mentioned previously, mass transfer performance of substances are mainly affected by their sizes, surface electrical properties and hydrophilic and hydrophobic properties. Zinc ion was a typical material on researches of transmembrane mass transfer. Methylene blue, because of its simple molecular structure, was often used as a representative of dye in various studies. They both had great differences in the size of the molecular structure. But they both possessed two positive charges on their surfaces. Neither methylene blue nor zinc ion cannot belong to biological macromolecules. It was quite meaningless to discuss their hydrophilic and hydrophobic properties. Using zinc ion and methylene blue molecule to study the transmembrane transport rules was representative. The mass transfer experiments of zinc chloride and methylene blue were simultaneously carried out under the same conditions of temperature and humidity. 

Acidic orange II molecule and methylene blue molecule are similar in size. Both of them had bright colors, which was beneficial for observation of experimental phenomena. It is known that methylene blue molecule was positively charged and acidic orange II molecule was negatively charged. Therefore, it is appropriate to investigate the effect of substance surface charge on transmembrane mass transfer using methylene blue molecule and acidic orange II molecule. The mass transfer experiments of methylene blue and acidic orange II were simultaneously carried out under the same conditions of temperature and humidity.

The intracellular fluid was simulated by zinc chloride solution, methylene blue solution and acid orange II dyeing liquid, respectively. The buffer or distilled water mimicked the extracellular fluid. At intervals, 1 mL samples were removed from the left and right sides of this experimental tank. A total of 6 samples were taken. Four parallel experiments were conducted for each substance. The concentration of different substances, transmitted to the extracellular environment, were measured at different times to explore the transmembrane transmission of the substances with different physical and chemical properties.

### 2.4. Biomimetic Cell Membranes Response to System Environment

#### 2.4.1. The Method of Adding Permeability Agents

In order to mimic intracellular fluid, 50 mL of methylene blue, acid orange II dye liquor, and nanosuspension of pigments were added to the left room, respectively. Meanwhile, 50 mL of distilled water was added into the right room as the extracellular fluid. In order to investigate the effect of permeability agents on transmembrane transport of different substances, 18 g/L Tween 80 and 20 g/L Triton X-100 were added to 50 mL of distilled water, respectively. Extracellular concentrations of different substances were changed with time. At intervals, 1 mL samples were removed from the left and right sides of this experimental tank. A total of 6 samples were taken. Four parallel experiments were conducted for each substance. 1 mL samples were diluted with water to 4 mL. Then, the absorbance of each sample was measured at the maximum absorption wavelength. The extracellular concentrations can be calculated according to the fitting straight line of concentration/absorbance. Then, the concentration of different substances transmitted to extracellular environment was plotted versus transport time.

#### 2.4.2. Physical Processing Method

To explore the effect of the physical processing method on transmembrane transport of different substances, the left room containing 50 mL of methylene blue, acid orange II dye liquid, or nanosuspension of pigments simulated intracellular fluid. The right room with 50 mL of distilled water was used as the extracellular fluid. Two graphite electrodes were fixed on the wall of both sides of the Plexiglas tank to study the effect of electric field on transmembrane mass transfer of different substances. The distance between the two electrodes was 10 cm. Two graphite electrodes respectively connected to the positive and negative electrodes of the DC regulated power supply with a voltage setting of 20 mV. The electrode connected to the positive electrode was on the left side of the experimental tank. In order to investigate the effect of magnetic field on transmembrane mass transfer, a 30 mT ferromagnetic sheet was pasted at the bottom of the Plexiglas tank. At intervals, 1 mL samples were removed from the left and right sides of this experimental tank. A total of 6 samples were taken. Four parallel experiments were conducted for each substance. The concentration of different substances transmitted were calculated with reference to the method of Section 2.4.1. The concentration of extracellular substances was plotted against the transmission time under the condition of the electric field and the magnetic field, respectively. 

### 2.5. Preparation of Pyrrole Structure Nano-Pigment by Biosynthesis

The pyrrole structure nano-pigments were prepared by the fermentation of *Serratia marcescens* in accordance with the previous paper [26]. The *Serratia marcescens* was cultivated in seed culture media at 30 °C and 160 rpm for 24 h in a shaking incubator and then inoculated into the fermentation culture media, followed by cultivation at 28 °C and 200 rpm for 72 h.

The seed culture: 5 g/L yeast powder, 10 g/L peptone, 3 g/L NaCl, 2 g/L KCl

The fermentation culture: 15 g/L peptone, 0.3% (*V*/*V*) glycerol, 2 g/L MgSO_4_, 3 g/L NaCl and 2 g/L KCl.

Upon in fermentation experiment, 1.8% (*V*/*V*) Tween 80, 2.0% (*V*/*V*) Trition x-100, the mixture of 0.9% (*V*/*V*) Tween 80 and 1.0% (*V*/*V*) Trition x-100 were added to the fermentation culture, respectively. Compared with the control group, experiment groups explored the environmental responsiveness of the fermentation preparation processes. At an interval of 12 h, 4 mL of the cultivated bacteria solution was taken from the fermentation medium, which was centrifuged at 9600 rpm, 10 °C for 10 min to discard the thalli and obtain nanosuspension of pigments. Then it was diluted 100 times by 90% acid ethanol-water solution (pH 3), which was subsequently a centrifuge for 5 minutes at 8000 rpm to remove insoluble matter sufficiently. Finally, a UV/visible spectrophotometer was used to measure the absorbance at 535 nm. The standard curve was generated at wavelengths of 535 nm for this pigment. The concentration of extracellular pigments was calculated according to the standard curve and plotted versus fermentation time.

## 3. Results and Discussion

### 3.1. Effect of Structure and Performance on the Transmembrane Transport of Substances

The issue of the mass transfer limitations could be further understood by the interaction of particles with phospholipid membranes [27,28,29]. The artificial membrane was disturbed or destroyed to varying degrees by different physicochemical properties of microparticles [30,31,32], especially the size of the particles [33,34,35]. Meanwhile, the ability of the particles to pass through the membrane was also affected by the surface chemical modification of nanoparticles [36]. This experiment mainly studied the effect of the size and surface electrical behavior of the substances on the transmembrane transport. 

#### 3.1.1. Effect of Substance Size on Transmembrane Mass Transfer

**Figure 3:**
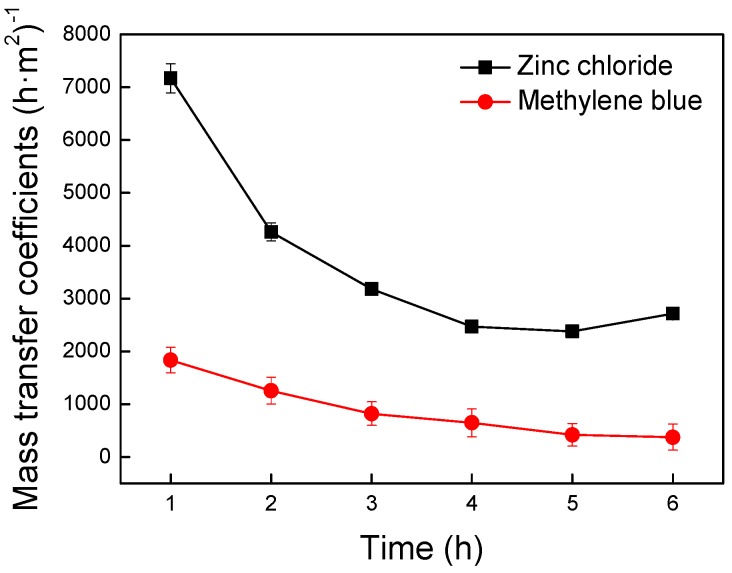
Effect of substance particle size on transmembrane mass transfer.

Zinc ion was a typical material on researches of transmembrane mass transfer. Methylene blue was the simplest dye molecule. They both had great differences in the size of the molecular structure. But they both possessed a positive two charge. Exploring the effect of molecular size on transmembrane mass transfer was fundamental for the subsequent studies. Using zinc ion and methylene blue molecule to study the transmembrane transport rules was representative. The volume of water-soluble molecules have been reported to be directly proportional to the relative molecular mass [37]. The relative molecular mass are 136.30 and 319.86 for the zinc ion and the methylene blue molecule, respectively. It has been apparent that the methylene blue molecule has a larger size than the zinc ion. To explore the effect of substance size on transmembrane mass transfer, the intracellular fluid was simulated by methylene blue dye liquor and zinc chloride solution, respectively. As shown in Figure 3, the mass transfer coefficient of that was plotted versus transfer times to investigate the transmembrane transmission of different substances.

The mass transfer coefficient is the important parameter for evaluation in mass transfer performance of different substances. Previous similar research on the interaction between microparticles and cell membranes found that the avenue of macromolecules excreted by cells were closely related to their geometric properties [38]. As shown in Figure 3, the mass transfer coefficient of zinc chloride was higher than methylene blue. This experiment indicated that the size of molecular structures had a significant effect on the transmembrane mass transfer. The smaller the molecular structures were, the easier they were to cross the membrane. Phospholipid molecules were not immobile in the membrane but were in a flowing state. Each phospholipid molecule in the membrane was surrounded by a different environment. Hence, the difficulty of the particles passing through the membrane was different depending on the position of the membrane surface. The higher lateral mobility of the particles was more advantageous to find the best location for penetration. The smaller particles showed the better migration ability, which led to the stronger membrane penetration. However, for the larger particles, the contact area with the membrane and driving force became higher the larger the size was. Therefore, the larger particles were confined to a certain part of the membrane with the reduction of migration activities and the increase of mass transfer resistance [38]. The smaller substances particle size, the easier they would pass though the membrane.

#### 3.1.2. Effect of Substance Surface Electrical Properties on Transmembrane Mass Transfer

**Figure 4:**
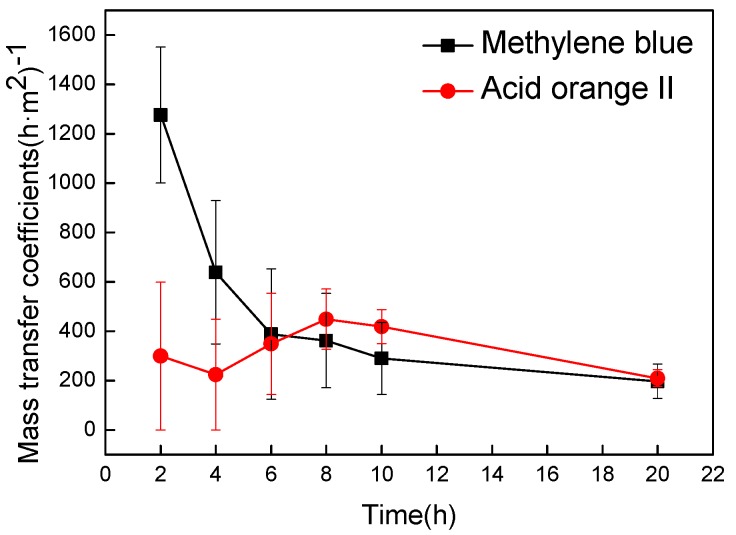
Effect of substance surface charge on transmembrane mass transfer.

The relative molecular mass are 319.86 and 350.32 for methylene blue molecule and acidic orange II molecule, respectively. Two dye molecules are similar in size. However, it is known that methylene blue molecules are positively charged and acidic orange II molecules are negatively charged. Therefore, it is appropriate to investigate the effect of substance surface charge on transmembrane mass transfer using methylene blue molecule and acidic orange II molecule. The intracellular fluid was simulated by methylene blue dye liquor and acidic orange II dyeing liquid, respectively. As shown in Figure 4, the mass transfer coefficients were plotted versus transfer times to illustrate transmembrane transmission of different substances. 

In the prophase of mass transfer, the mass transfer coefficient of methylene blue with positive charge was significantly higher than acid orange II with negative charge (Figure 4). A positively charged substance had a preferential attraction and a higher chance of penetration for cell membrane due to the electric interaction between positively charged substances and negatively charged glycocalyx on the cell membrane. In contrast, the negatively charged acid orange II molecule had been a repulsion for the cell membrane, which caused a lower probability of transfer. Therefore, the mass transfer rate of positively charged substances were faster than other substances [39,40,41,42,43,44,45]. In the late stage of the transfer experiment, the mass transfer coefficients of the two substances were very close. The transfer was close to the dynamic balance because the concentration difference between inside and outside the membrane was quite slight. At this time, the transmembrane transport was mainly affected by the mass transfer driving force. The total mass transfer effect of methylene blue was clearly better than acid orange II (Figure 4), indicating that the positively charged substances have better mass transfer effect than the negatively charged.

### 3.2. Transmembrane Mass Transfer Response to the Environment

The permeability of the biofilm can be affected by the liquid environment surrounding the membrane. Some changes of the surroundings can improve the cell membrane permeability, which contribute to penetrating the cell membrane for the larger particles [26,46]. General methods for improving cell membrane permeability include the way of adding permeability agent and physical treatment [47]. In this experiment, the mass-transfer effect was enhanced using such surface-active permeability agents as Tween 80 and Trition X-100. Physical treatments such as electric field and magnetic field were carried out to increase the permeability of the bilayer lipid membrane.

#### 3.2.1. Permeability Agents Regulate Transmembrane Mass Transfer

**Figure 5:**
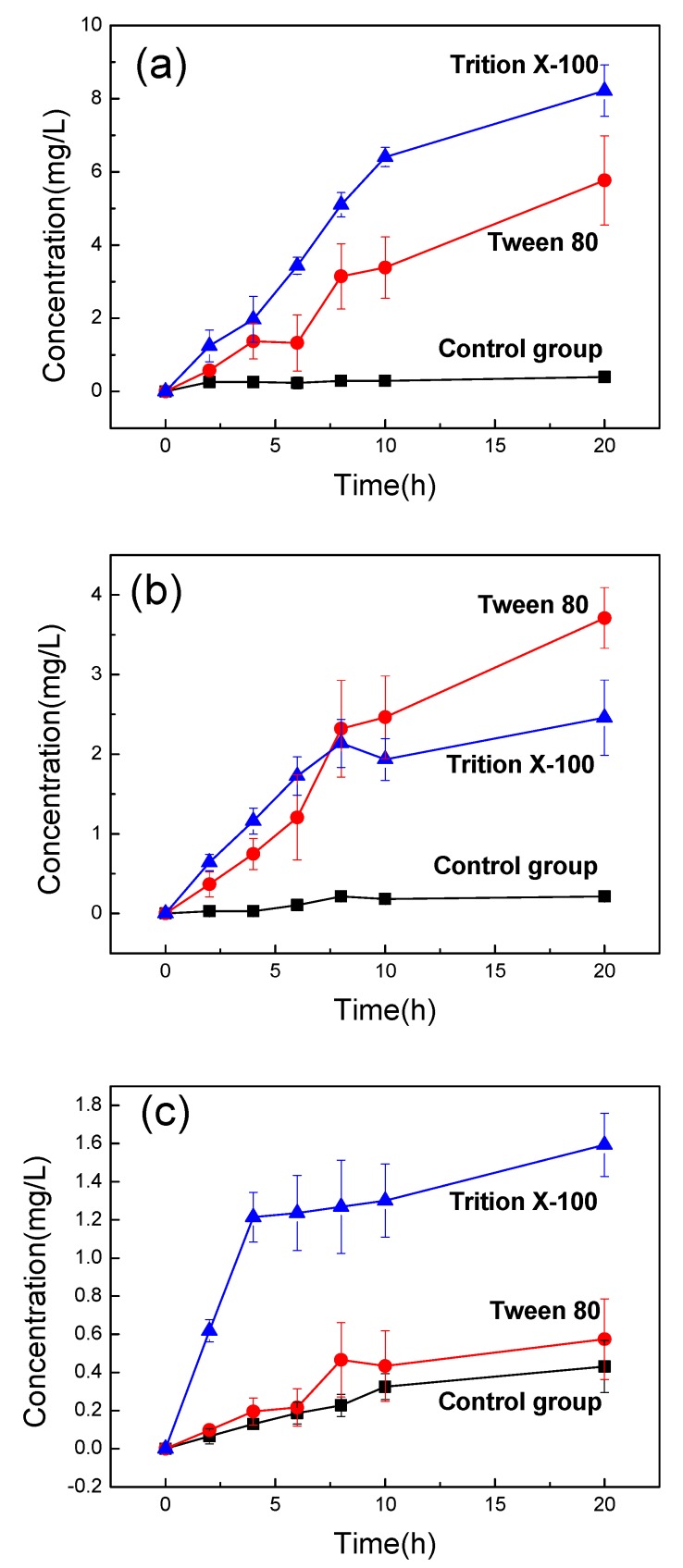
Change of extracellular concentration of different substances with time: (**a**) Methylene blue, (**b**) Acid orange II, and (**c**) Pyrrole structure nano-pigment.

This experiment used Tween 80 and Trition X-100 to determine the effect of permeability agents on membrane permeability according to previous literature [48,49]. The intracellular water was mimicked by methylene blue dye liquor, acidic orange II dyeing liquid, and nanosuspension of pigments, respectively. The permeability agents were added to the extracellular fluid to adjust the barrier of the bilayer lipid membrane and improve membrane permeability. As shown in Figure 5, the extracellular concentrations of that were plotted versus transfer times to study the transmembrane transmission of different substances. 

The mass transfer effect of the three experimental groups with adding permeability agents were clearly improved (Figure 5). As shown in Figure 5a,c, the improvement of mass transfer resulted after addition of Trition X-100 were found to be more pronounced than those after the addition of Tween 80. Figure 5b shows that both Tween 80 and Trition X-100 could promote the mass transfer significantly with differences not being obvious. This is mainly because both types of both permeability agents contain long chains of hydrophilicity and hydrophobicity. The hydrophobic part could be inserted into the lipid membrane. The hydrophilic group can be embedded in the bilayer and protrude into the aqueous phase. Meanwhile, phospholipid molecules are not immobile in the cell membrane, also known as “the fluid mosaic model”, which can increase the fluidity and permeability of the lipid membrane and improve the proportion of extracellular pigment.

When the permeability agents were added, the extracellular pigment concentration of the methylene blue was increased by a factor of 20 (Figure 5a). The best effect of the acid orange II was approximately a 17-fold higher compared to the control group (Figure 5b). Whereas, for pyrrole structure nano-pigment, the extracellular concentration was merely increased up to four times by using permeability agents (Figure 5c). The pyrrole structure nano-pigments with poor dispersibilities were mainly present in the form of nanoscale particles in solution because they were almost insoluble in water at room temperature. In contrast to methylene blue and acid orange II, pyrrole structure nano-pigment possessed a larger size. The larger volumes of substances tended to increase the resistance to motion within the phospholipid bilayers thus a lower mass transfer rate could be exhibited.

#### 3.2.2. Physical Treatments Regulate Transmembrane Mass Transfer

**Figure 6:**
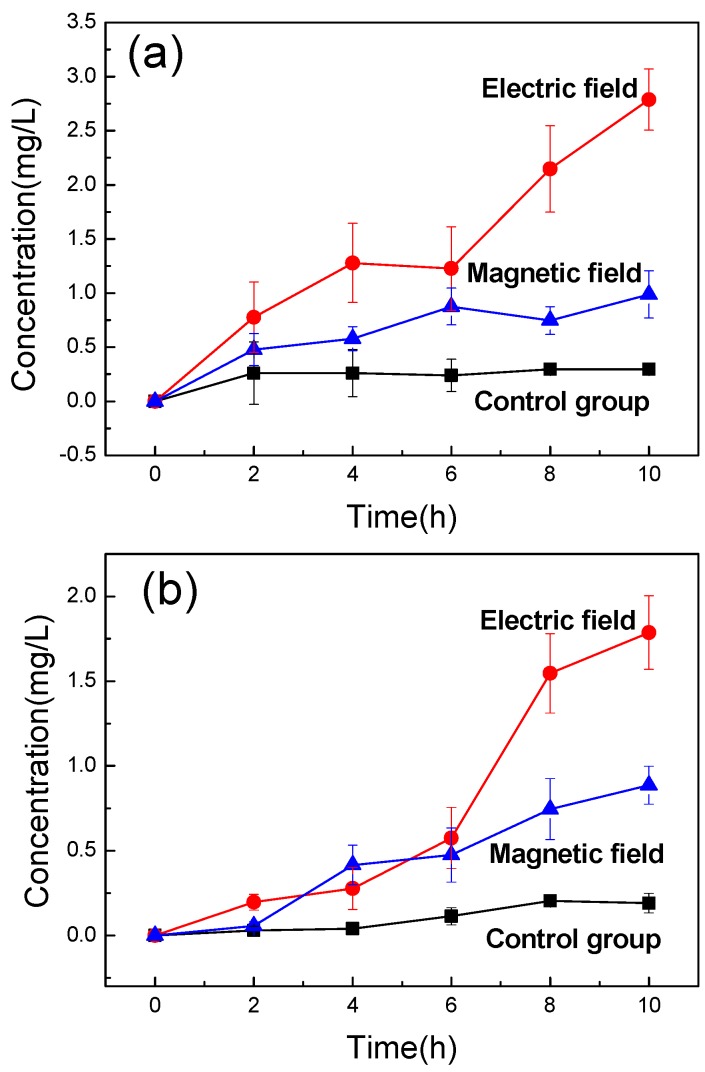

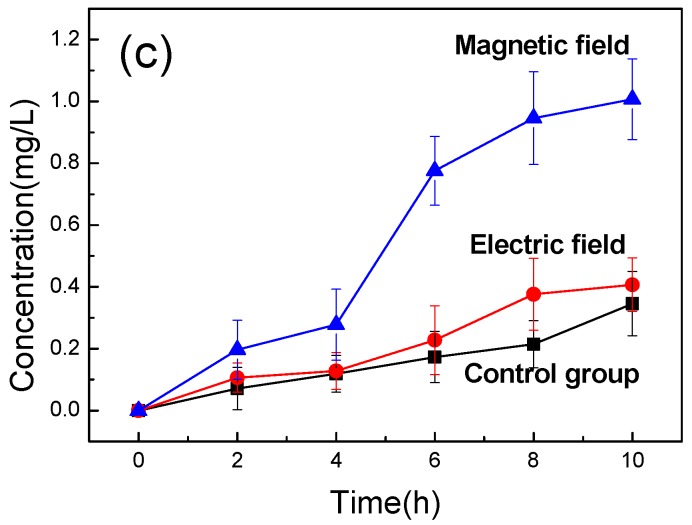
Change of extracellular concentration of different substances with time: (**a**) Methylene blue, (**b**) Acid orange II, and (**c**) Pyrrole structure nano-pigment

It was reported that the permeability of the lipid membrane can be improved by physical treatment [47]. To explore the effect of physical treatment methods on membrane permeability, the transport experiments of different substances were carried out under the conditions of electric field and magnetic field, respectively. As shown in Figure 6, the extracellular concentration of different substances were plotted versus transfer times to study the transmembrane transmission.

The mass transfer effects of the three substances were all improved after physical treatment (Figure 6). This is mainly because the particles would move toward the opposite charge when the charged substances were subjected to an electric field or magnetic field. The mass transfer effect have been enhanced to varying degrees by different traction. The improvement effect of the electric field was superior to the magnetic field as shown in Figure 6a,b, Whereas the improvement effect of magnetic field was better than electric field in Figure 6c. Specifically, the mass transfer effect is mainly affected by the electric field force generated by electric field since methylene blue and acid orange II were charged. However, the existence state of the pyrrole structure nano-pigment was in the form of a suspension with the surface uncharged, which caused the mass transfer effect to be mainly affected by the magnetic field. The initial action site of the magnetic field was the cell membrane, which could change the charge distribution of the membrane surface and promote the fluidity of the cell membrane. Thus, the permeability of the cell membrane was improved and the proportion of extracellular pigment was increased.

After the physical treatment, the extracellular concentration of the methylene blue was increased by a factor of nine (Figure 6a). The best enhancement effect of the acid orange II was also approximately nine-fold higher compared to the control group (Figure 6b). Yet, the best improvement effect of the pyrrole structure nanopigment was only about three times higher than that of the control group (Figure 6c). This result was mainly attributed to the physicochemical properties of the pigments. The pyrrole structure nano-pigments, poorly soluble in water, mainly existed as nanoscale particles in aqueous solution due to its poor dispersity. Furthermore, the size of the pigment was larger than the methylene blue and acid orange II. The larger volumes of substance tended to increase the resistance to motion within the lipid bilayer membrane, thus a lower mass transfer rate would be exhibited.

### 3.3. Preparation and Regulation of Pyrrole Structure Nano-Pigment by Biosynthesis

**Figure 7:**
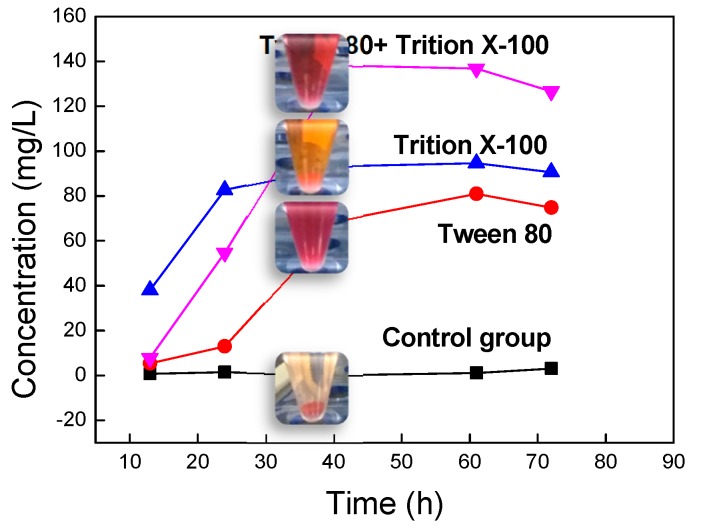
Change of pigments output with time.

In this study, a model membrane platform was established with a planar lipid bilayer to investigate the biological preparation of microbial pigments and explore the breakthrough methods of cell membrane barrier. To demonstrate the results of previous simulation experiment, the permeability agents were added to the biological preparation of pyrrole structure nano-pigment. The amount of extracellular pigment growth plotted versus fermentation times (Figure 7).

As shown in Figure 7, the improvement effect exhibited a substantial higher level of Trition X-100 as compared with other experimental groups between 12 and 24 h. This is mainly because Trition X-100 could directly interact with lipids of cell membrane, which rapidly and significantly improved the permeability of cell membrane. Moreover, Trition X-100 could cause a large increase in the biomass of *Serratia marcescens* in a short time. However, the biomass of *Serratia marcescens* with Tween 80 was still less in a shorter period of time. A large number of microorganisms produced more secondary metabolites, which was beneficial to improve the extracellular concentrations of pigment. Tween 80 had a significant effect in 24–48 h. When both permeability agents were added simultaneously, the amount of extracellular pigments growth considerably increased from 12 h to 48 h. This is because the effect of the permeability agents on cell membrane permeability. The results of the simulation experiment of the planar bilayer lipid membrane were verified. The extracellular pigment concentrations in the four groups did not have great differences from 48 h to 60 h. The highest yield with Tween 80 & Trition X-100 appeared around 40 h. Overall, the concentrations of the four groups reached or approached the maximum at 60 h. This is mainly determined by the growth cycle of *Serratia marcescens* and the production time of pigments under specific culture conditions. The extracellular concentrations were decreased slightly in three experimental groups because the nutrients in the fermentation broth gradually decreased with culture time. A small amount of pyrrole structure nano-pigments released to the extracellular were consumed by the growth of bacteria. When two permeability agents were used separately, Trition X-100 performs better effect than Tween 80 (Figure 6). To a certain degree, our experimental results were complementary to previous simulation studies. After 72 h, the maximum concentration of extracellular pigments in the experimental groups was 126.68 mg/L, which was 40 times higher than the control group of 3.13 mg/L. This result indicated that the synergistic effect of the two permeability agents produced the best enhancement. In such case, the amount of extracellular pigment growth was significantly higher than the respective agents used alone.

## 4. Conclusions

The sizes and surface electrical properties of the substances have contributed much to the transmembrane transport of substances. The smaller volumes of substances tended to decrease the resistance to motion within the phospholipid bilayer, which was beneficial for the transmembrane transport. Furthermore, mass transfer effect of positively charged substances were better than that of negatively charged substances. The system environment also had an important impact on the transmembrane transport of substances. Both the methods of adding permeability agents and the physical treatment can improve the permeability of the cell membrane and the effect of the former method was more obvious. Trition X-100 added to extracellular water was more conducive to mass transfer across the membrane, when only one type of permeability agent was used. Compared with the applied magnetic field, the applied electric field was favorable to improve membrane permeability and boost extracellular substance concentration. Overall, based on the results of simulation experiment of the lipid bilayers, the cell membrane permeability agents were added to the fermentation culture of pyrrole structure nano-pigment. This result indicates that in the case when the only one type of permeability agent is used, addition of Trition X-100 resulted in better improvement of permeability than Tween 80 did. Furthermore, the synergistic effect of the two permeability agents produced the best improvement effect. In this case, the amount of extracellular pigment growth was significantly higher than that used alone. This research has realized the efficient preparation of microbial dye liquor based on fermentation broth and promoted the application and development of microbial pigments.
